# Nephrotic syndrome presented as a portal vein thrombosis: a case report

**DOI:** 10.1097/MS9.0000000000000482

**Published:** 2023-04-06

**Authors:** Mohamed Osman Omar Jeele, Abdisamad M. Adan

**Affiliations:** Department of Internal Medicine, Mogadishu Somali Turkish Training and Research Hospital, Mogadishu, Somalia

**Keywords:** abdominal pain, hypoalbuminemia, nephrotic syndrome, portal venous thrombosis

## Abstract

Nephrotic syndrome (NS) is a clinical disorder characterized by significant proteinuria, hypoalbuminemia, hyperlipidemia, edema, and other complications. Urinary loss of clotting inhibitors, zymogens, and plasminogen; increased hepatic synthesis of fibrinogen and lipoproteins, and hemoconcentration due to fluid loss are some of the factors which predispose NS patients to hypercoagulable states like portal vein thrombosis.

**Case presentation:**

In the presenting case report, we described a 21-year-old woman with no history of NS and a hypercoagulable state who presented to our emergency department with severe generalized abdominal pain and lower limb edema. She was subsequently diagnosed with NS complicated with portal vein thrombosis and was admitted to our internal medicine unit. After 2 weeks of treatment, the patient was discharged in good health.

**Conclusion:**

Additional evaluation for newly onset NS with venous thrombosis should be needed in the presence of severe abdominal pain and lower limb edema even in a patient without a previous history of NS.

## Introduction

Highlights

- Nephrotic syndrome (NS) is a clinical disorder characterized by significant proteinuria, hypoalbuminemia, hyperlipidemia, edema, and other complications.
- Sustained hypoalbuminemia is attributed to hypercoagulability and a higher risk of venous thromboembolism (VTE).
- The incidence of portal vein thrombosis (PVT) in individuals with NS is quite low.

NS is a clinical disorder characterized by significant proteinuria, hypoalbuminemia, hyperlipidemia, edema, and other complications^1^. It is caused by impairment in glomerular permeability, which may be due primarily to intrinsic renal pathology or secondarily to various causes such as congenital causes, infections, diabetes, systemic lupus erythematous, neoplasia, or the use of drugs^2^. It has been observed that VTE including pulmonary embolism, deep venous thrombosis, renal vein thrombosis, and inferior vena cava thrombosis are the most common life-threatening complications of NS^3^. PVT is thrombosis that occurs in the body of the portal vein and also its right and left intrahepatic branches and may spread to the splenic or superior mesenteric veins. PVT may be related to cirrhosis or cancer of the liver, or it may develop independently of liver pathology, such as in cases of NS^4^. The incidence of PVT in individuals with NS is quite low^5^. Although there are some cases around the world which report the occurrence of PVT in patients with NS, there is no previous case reported from Somalia regarding this subject. In the presenting case report, we describe a 21-year-old woman who presented to the emergency department complaining of generalized abdominal pain and was later diagnosed with PVT due to NS.

## Case report

A 21-year-old woman, previously a healthy, nonsmoking woman, with no known history of surgical and chronic diseases, presented to our emergency department with a complaint of severe generalized abdominal pain associated with nausea and vomiting for 3 days and lower limb edema for 3 weeks. She had no history of similar conditions. Her family history was unremarkable. On examination, she looked ill; she had a swollen face and was in some distress. Physical examination of the cardiovascular, respiratory, and nervous systems was unremarkable. Her abdomen was mildly distended; she had diffuse pain and rebound tenderness in her abdomen; and she had significant guarding around her abdomen. There was no evidence of organomegaly. Bowel sounds were normal on auscultation of the abdomen. The patient had bilateral grade 2 lower limb edema reaching the knees. Her vital signs were as follows: pulse 70 bpm, blood pressure 110/80, respiratory rate 16, and temperature 36.9°C.

**Figure 1:**
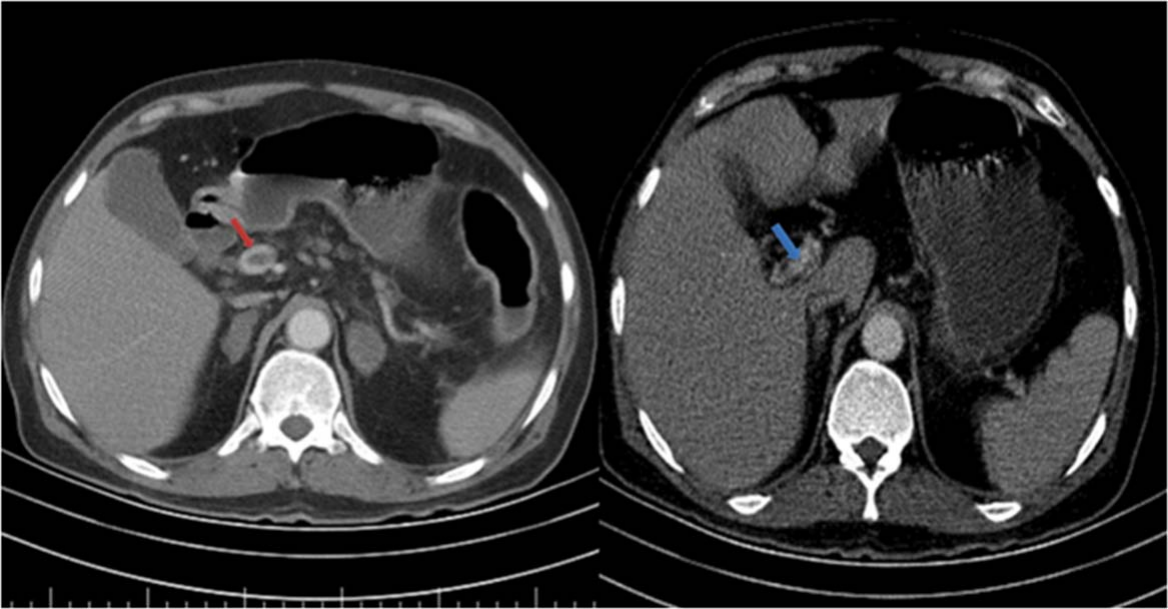
Axial contrast-enhanced abdominal computed tomography examination shows filling defects in the lumen of the portal vein, suggesting portal vein thrombosis. Red arrow shows arterial phase and blue arrow shows venous phase

Laboratory investigations revealed a hemoglobin level of 12.5 g/dl, aspartate aminotransferase 87 U/l, alanine aminotransferase 102 U/l, urea of 56 mg/dl, creatinine of 1.5 mg/dl, serum sodium 132 mmol/l, and potassium of 3.72 mmol/l. Total protein was 3.99 g/dl, albumin was 1.88 g/dl, total cholesterol was 310 mg/dl, and low-density lipoprotein was 218 mg/dl, prothrombin time 14 s, international normalized ratio 0.9, fibrinogen 2.02 g/l (normal range: 2–4 g/l), d-dimer 2.5 mcg/ml (normal range: 0–0.5 mcg/ml), and activated partial thromboplastin time 24 s. Protein was detected in urinalysis, and the urine protein-to-creatinine ratio resulted in 14.27 g/day. A chest radiograph revealed mild pleural effusion. Abdominal doppler ultrasound was performed and showed features of PVT. An abdominal computed tomography scan with contrast enhancement was ordered and showed filling defects in the lumen of the portal vein, suggesting PVT (Fig. 1). On the basis of the above-mentioned results, the patient was admitted to our internal medicine unit with the established diagnosis of NS complicated by PVT. She was started with methylprednisolone, ramipril, atorvastatin, and furosemide, and low–molecular-weight heparin. After 2 days, warfarin was added to her regimen. Her symptoms improved after 1 week of hospitalization; her abdominal pain and lower limb edema decreased. After 2 weeks of treatment, her urine protein-to-creatinine ratio was decreased to 5 g/day and she was discharged in good health. The patient had returned for her routine follow-up 3 weeks later after discharge, and her general health condition was very good. She did not have any abdominal pain, and her urine protein-to-creatinine ratio was 1.8 g/day. The follow-up abdominal ultrasound did not reveal any thrombus or dilated portal vein.

## Discussion

Since 1840, venous thrombosis has been known as a complication of NS, although the mechanism behind this consequence has only been identified more recently^6^. Urinary loss of clotting inhibitors, zymogens, and plasminogen; increased hepatic synthesis of fibrinogen and lipoproteins, and cofactors such as factors V, VII, and VIII; excessive platelet aggregation; hemoconcentration due to loss of intravascular fluid; and the side effects of drugs (diuretics and steroids) have been related to causing this complication^6^. Also, sustained hypoalbuminemia is attributed to hypercoagulability and a higher risk of VTE^5^. It’s believed that around 25% of NS will eventually suffer from thromboembolic complications^7^. Despite the fact that PVT can occur in NS patients, it is uncommon and typically develops after therapy or a recurrence of the disorder, rather than as the first symptom^8^.

The pathogenesis of PVT comprises one or more of Virchow’s triad, which consists of diminished portal blood flow, a hypercoagulable condition, and vascular endothelial damage^9^. The reported lifetime risk of PVT in the general population is 1%^10^.

In our case report, we described a 21-year-old woman who presented with NS and PVT at the same time without a previous history of NS and proteinuria. The patient initially presented with generalized abdominal pain and lower limb edema, and after investigation, was admitted to our internal medicine department with the diagnosis of NS complicated with PVT. The presentation of PVT along with the new onset of NS makes our case quite rare. In 2008, Sun and Xu^8^ reported a 52-year-old man who presented with PVT as the initial symptom of NS. In a similar manner, Wang and colleagues described a case of an 18-year-old male student with newly diagnosed NS who presented with portal, splenic, and superior mesenteric vein thrombosis. They concluded that thrombus can arise in the earliest stages of NS and in rare locations^11^. In addition, Park and colleagues documented the case of a 31-year-old man with a known history of NS owing to minimal change disease and who has a history of remissions and relapses. Their patient presented with generalized abdominal pain and was diagnosed with venous thromboembolism in the portal, spleen, and superior mesenteric veins as a complication of NS^12^.

This study has been reported in line with the SCARE 2020 criteria^13^.

## Conclusion

The presence of severe abdominal pain and lower limb edema in a patient without a previous history of NS may warrant additional evaluation for newly onset NS with venous thrombosis in rare locations such as the portal vein.

## Ethical approval and consent for publication

Mogadishu Somali Turkish Training and Research Hospital ethics committee waived approval for this case report.

## Consent

Written informed consent was obtained from the patient for publication of this case report. A copy of the written consent is available for review by the Editor-in-Chief of this journal on request.

## Sources of funding

The authors declare no funding source.

## Author contribution

All authors contributed to this manuscript substantially whether the conception, it’s drafting, and revising the final manuscript.

## Conflicts of interest disclosure

The authors declare that they have no financial conflict of interest with regard to the content of this report.

## Research registration unique identifying number (UIN)

1. Name of the registry: NA
2. Unique Identifying number or registration ID: NA
3. Hyperlink to your specific registration (must be publicly accessible and will be checked): NA

## Guarantor

Mohamed Osman Omar Jeele.

## Provenance and peer review

Not commissioned externally peer-reviewed.
